# Singing modulates mood, stress, cortisol, cytokine and neuropeptide activity in cancer patients and carers

**DOI:** 10.3332/ecancer.2016.631

**Published:** 2016-04-05

**Authors:** Daisy Fancourt, Aaron Williamon, Livia A Carvalho, Andrew Steptoe, Rosie Dow, Ian Lewis

**Affiliations:** 1 Centre for Performance Science, Royal College of Music, London SW7 2BS, UK; 2 Faculty of Medicine, Imperial College, London SW7 2AZ, UK; 3 Psychobiology Group, Department of Epidemiology and Public Health, UCL, London WC1E 6BT, UK; 4 Tenovus Cancer Care, Gleider House, Ty-Glas Rd, Cardiff CF14 5BD, Wales, UK

**Keywords:** singing, cancer, cortisol, oxytocin, cytokine, beta-endorphin, inflammation, stress, mood, music

## Abstract

There is growing evidence that psychosocial interventions can have psychological benefits for people affected by cancer, including improved symptoms of mental health and wellbeing and optimised immune responses. However, despite growing numbers of music interventions, particularly singing, in cancer care, there is less research into their impact. We carried out a multicentre single-arm preliminary study to assess the impact of singing on mood, stress and immune response in three populations affected by cancer: carers (n = 72), bereaved carers (n = 66) and patients (n = 55). Participants were excluded if pregnant or if they were currently being treated with chemotherapy, radiotherapy or oral immunosuppressive drugs. Participants were regular participants in five choirs across South Wales and took part in one hour of group singing. Before and after singing, visual analogue mood scales, stress scales and saliva samples testing for cortisol, beta-endorphin, oxytocin and ten cytokines were taken. Across all five centres and in all four participant groups, singing was associated with significant reductions in negative affect and increases in positive affect (p < .01) alongside significant increases in cytokines including GM-CSF, IL17, IL2, IL4 and sIL-2rα (all p < .01). In addition, singing was associated with reductions in cortisol, beta-endorphin and oxytocin levels. This study provides preliminary evidence that singing improves mood state and modulates components of the immune system. Further work is needed to ascertain how this differs for more specific patient groups and whether repeat exposure could lead to meaningful, longitudinal effects.

## Background

Over the past 50 years, more than 300 studies have explored the combined psychological and biological value of psychosocial interventions, from mindfulness to yoga to the arts, for cancer patients, survivors and carers [1–3]. Psychosocial interventions for all three populations have been found to reduce symptoms of depression and anxiety, increase social support networks, improve quality of life and raise perceptions of care [2]. These positive states have, in turn, been linked to optimised immune responses including the lowering of inflammation, enhanced cellular function and other longer term health outcomes [4–5]. In contrast, negative psychological states and limited social interactions have been linked to lower white blood cell activity, reduced numbers of antibodies and increased stress hormone response [2, 4–5]. Such evidence suggests that psychosocial interventions could play an important role in optimising health in people affected by cancer, helping to put patients in the best position to receive treatment or maintain remission and supporting staff and relatives who care for someone with cancer.

Specifically, there has been a growing interest in the role of music as one such psychosocial intervention. Thirty randomised controlled trials were included in a Cochrane review on music and cancer [6] ranging from music therapy sessions to listening to recorded music. Studies have shown reductions in anxiety, improvements in mood and reductions in cardiovascular measures such as blood pressure. However, to date there has been little attention on the impacts of music interventions on biological markers of endocrine and immune function. There is nevertheless evidence to suggest that music interventions could have a combined effect on the mental health and immune function of people affected by cancer. Biobehavioural research has proposed that three key components are important in achieving immune changes in cancer patients and carers: emotional expression, benefit finding and social support [2]. Music interventions in cancer care are therefore a pertinent avenue to explore as they fulfil all three components: they have the potential to help participants express their emotions, enable them to interact with music as a new hobby and are inherently social. There is also growing evidence of the impact of music-making on neuroendocrine and immune responses in other patient populations [7], with previous studies showing that singing in particular can reduce levels of stress hormones such as cortisol, cortisone and progesterone and increase social bonding including the release of oxytocin [8–11]. Finally, animal research has shown that music played to rodents reduces the suppressive immune effects of stress associated with cancer, enhances anti-tumour response and improves white blood cell function [12], further paving the way for biological studies in humans.

Within service development, there are a growing number of organisations delivering singing programmes for people affected by cancer, including patients, carers and staff. One example of this is the work of Tenovus Cancer Care in Wales, whose choirs involve over 1,000 people affected by cancer singing every week. Research around these choirs has identified that long-term involvement across three and six months is associated with reduced levels of anxiety and depression and improved quality of life [13]. Singing has also been found to provide support and processing of grief for cancer patients and their families [26]. However, it remains unknown whether, parallel to psychosocial benefits, singing could have effects on immune response.

Consequently, this study aimed to explore whether people affected by cancer who sing regularly in choirs experience psychobiological responses to singing across a single choir session. As this study was the first of its kind, it was conducted as a preliminary single-arm study with two main aims: (i) to compare changes across time in three separate populations affected by cancer: current carers, bereaved carers and patients; (ii) to assess whether responses from the three groups differed from one another in order to explore whether singing was of specific value for any particular group.

## Methods

### Design and participants

This was a multi-centre single-arm study comparing responses between three participant groups. In order to make sure that findings were not just specific to a single choir, five choirs were approached via convenience sampling from a total of 18 choirs for people affected by cancer run by Tenovus Cancer Care in Wales. Participants from within these choirs were invited to take part and recruited at the sites where they attended the choirs. A total of 251 patients were screened and 193 were found to be eligible and consented. Participants were categorised into four groups: current carers of someone with cancer (n = 72), bereaved carers (n = 66), cancer patients (n = 55). For all three groups, participants had to have attended at least 1 choir session and were excluded if they were under 18 years of age. Cancer patients either had a current diagnosis of cancer or were in remission and were excluded if they were currently registered on another clinical trial, if they had a diagnosis of mouth cancer as this would affect saliva samples, if they were pregnant, if they were currently undergoing any active cancer treatment including chemotherapy or radiotherapy, or if they were taking oral immunosuppressive drugs. The study protocol was approved by the Conservatoires UK Research Ethics Committee and all participants gave written informed consent prior to the study.

### Procedure

Participants took part in a single 70-minute choir rehearsal between 19.00 and 20.10 led by a trained choir leader at the usual community locations used each week as sites for the five choirs. Sessions consisted of warm-up exercises, learning new songs as a group and singing songs already in the singers’ repertoire. The week preceding the rehearsal, participants were asked to fill in a pack of demographic and psychological scales, then immediately before and after the rehearsal, participants gave a saliva sample and filled in a set of visual analogue scales assessing mood and stress.

### Psychological measures

Participants’ socio-demographic and health characteristics were obtained by means of a set of self-administered questionnaires. This included assessment of wellbeing using the Warwick-Edinburgh Mental Wellbeing Scale (WEMWBS); a questionnaire used extensively as a measure of mental health ranging from 14–70 with higher scores indicating better wellbeing [15]; anxiety and depression with the Hospital Anxiety and Depression Scale (HADS) [16], ranging from 0–21 for each construct with higher scores indicating poorer mental health; and social function with the Connor-Davidson Resilience Scale (CD-RISC) ranging from 0–100 with higher scores indicating stronger resilience [17].

Visual analogue scales measuring 12 different moods were also assessed. These consisted of two specific sets of visual analogue scales plus an additional measure. One set asked participants to rate specified moods on a series of 100 mm lines (higher responses indicating more intense moods): sad, afraid, angry, tired, energetic, happy and confused [19–21]. Another set assessed participants’ anxiety, relaxation and stress levels [22]. A final visual analogue scale assessed social connectedness. This provided a total of 12 measures of moods.

### Biological measures

Given recent research suggesting the promise of saliva as an alternative to blood analysis in biobehavioural research [23–25], this study used the technique of multiplex saliva assays for the assessment of cortisol and cytokines. Saliva was collected via a passive drool method facilitated by polypropylene straws into low-bind polypropylene 2 mL cryovials (Eppendorf, UK). Samples were taken at 7 pm (pre-sample) and 8.15 pm (post-sample). Samples were stored at -20°C for a period of 2 weeks before transfer to -40°C for one month prior to analysis at Aeirtec Laboratories. Samples were vortexed and centrifuged before analysis. Supernatants were pipetted into 96 well filter plates (Millipore, UK) with microplex_TM_ beads (Luminex Corp, USA) conjugated with antibodies to the analytes. Following research showing that both acute and chronic stress and depression are associated with increases in stress hormones and inflammatory immune response [29–30], the samples were analysed primarily for the neuroendocrine stress hormone cortisol and six cytokines (including pro-inflammatory cytokines interleukin 2 (IL2), interferon gamma (IFNɣ) and tumour necrosis factor alpha (TNFα), anti-inflammatory cytokine IL4, and cytokines that display both pro- and anti-inflammatory profiles IL6 and IL17) and the pro-inflammatory chemokine monocyte chemoattractant protein 1 (MCP1). These markers have been shown to be responsive to both short and long-term psychosocial interventions, including music, in previous studies [27]. In addition, we included two soluble receptors, soluble interleukin 2 receptor alpha (sIL2Rα) and soluble tumour necrosis factor receptor 1 (sTNFr1), as well as the stem-cell differentiator growth factor granulocyte macrophage colony stimulating factor (GM-CSF) as indicators of cellular involvement. And, we also included the neuropeptides oxytocin and β-endorphin which have been implicated in social bonding and feelings of elation respectively [33–34]. All capture and detection antibodies and standards were purchased from Perotech, UK, with the exception of TNF-α (R&D Systems, UK) and cortisol (antibodies from Abcam, UK, tracer from Randox, UK, and standards from Sigma-Aldrich, UK). Following incubation for 24 hours, the beads were washed by filtration and then biotin-conjugated antibodies to the analytes were added to the beads for 4 hours. Following incubation with detection antibodies, the beads were washed and incubated with streptavidin-conjugated R-phycoerythrin for 45 minutes to provide a fluorescent detection signal. Following washing, the beads were analysed on a Luminex 100_TM_ analyser. The inter-assay coefficient of variation range for all analytes was 1.8–5.37%, and the intra-assay coefficient of variation range was 0.8–3.58%.

### Statistical analysis

Statistical analysis was performed using SPSS (Version 21.0, SPSS Inc, USA). A one-way analysis of variance (ANOVA) was used to test for differences in baseline demographic data between choirs and between patients versus carers. Repeated measures ANOVAs were used for psychological and biological data to test across time (pre- vs post-singing) and between groups (carers vs bereaved carers vs patients). The distribution of all biomarkers was positively skewed, and so the data were logarithmically transformed. As there were 12 mood states and 13 biomarkers assessed (a total of 25 markers), the significant p value for pre-post comparisons using Bonferroni’s adjustment was α < .002.

In addition to measuring mood through the eight components of the visual analogue mood scale, negatively-scored mood ratings were reverse scored and Cronbach’s alpha was calculated using all eight mood states to reflect a single aggregate measure of mood with higher levels indicating more positive mood. Similarly, the three components of the visual analogue stress scale were reverse scored and combined onto a single aggregate measure of mood, with higher levels indicating higher stress.

Correlations between psychological variables and between biomarkers respectively were made using Pearson’s product moment correlation coefficient. For interactions between psychological and biological data, Pearson’s product moment correlation coefficients were run and, where significant, regression models were developed with adjustments made for age and sex. Some biomarkers fell below the levels of detection during analysis which accounts for the adjusted degrees of freedom.

## Results

### Descriptive statistics

One hundred and ninety three participants took part in the study, with the majority being white women (Table 1). Participants across both groups were on average not symptomatic of having depression or anxiety and had average levels of wellbeing [8–11]. However, their social resilience scores were considerably lower than the general population (cf. 80.7) [17]. One-way ANOVAs revealed there were no significant differences in demographic data between the groups.

### Psychological results

#### Across time

There were no significant differences in mood at baseline between the three groups. Across a single choir session, there were significant effects of time across all twelve moods assessed, with increases in positive affect and decreases in negative affect (all p < .001) (Table 2). Exploratory analyses showed that these results were found consistently for all 5 choirs with no statistical difference in response.

Responses to all eight moods were correlated, as were responses to the three stress items, so for both, negatively scored constructs were reverse scored and aggregate mood and stress scales were constructed. For the aggregate scale for mood, Cronbach’s alpha was 0.83 for ‘pre’ measurements and 0.82 for ‘post’ measurements and for the aggregate scale for stress, Cronbach’s alpha was 0.77 for both ‘pre’ and ‘post’ measurements indicating that the component ratings were reliably interrelated. Aggregate mood was found to improve across the choir session (p < .001) and aggregate stress was found to decrease (p < .001).

Mood was particularly found to increase for those who had lower mental wellbeing, with significant positive correlations between changes in mood and baseline anxiety levels (r = .261, p < .001) and depression levels (r = .314, p < .001), and significant negative correlations between changes in mood and baseline levels of both wellbeing (r = -.222, p = .002) and social resilience (r = -.180, p = .013). Furthermore, there was a significant negative correlation between baseline mood and change in mood score across the intervention (r = -.680, p < .001). Similarly, stress was found to decreases particularly for those with higher anxiety and depression levels (r = -.273, p < .001 and r = -.267, p < .001). However, there was less of an association shown with positive measures of wellbeing, with no significant correlation with social resilience and only a very small correlation with wellbeing (r = .154, p = .034). As with mood, participants with higher levels of stress at baseline experienced the greatest changes during singing (r = -.789, p < .001).

#### Between groups

There were no significant differences between the three groups in individual or aggregate measures of mood, stress or connectedness, indicating a general consistency of effect (Table 2).

### Biological results

#### Across time

There were no significant differences in biological response at baseline between the three groups. Across a single choir session, there were significant effects of time for twelve of the thirteen biomarkers assessed; ten of which held when controlling for multiple comparisons with α < .0031 (Table 3). Exploratory analyses showed that these results were found across all 5 choirs with no statistical difference in response.

Overall, across all three groups, the results showed a significant decrease in cortisol and neuropeptide levels accompanied by an acute increase in cytokine and receptor activity (GM-CSF, IL17, IL2, IL4, TNFα, sIL-2rα and sTNFr1) suggestive of a general activation of the cytokine network. However, IFNγ and IL6 did not fall below the adjusted significance value of p < .002, and there was also no significant change over time for MCP1.

Change in cortisol had a medium-sized negative correlation with changes in IL2 (r = -.485, p < .001), TNF-α (r = -.326, p < .001), sIL2rα (r = –.339, p < .001) and IL4 (r = -.458, p < .001) and a small-sized negative correlation with changes in sTNFr1 (r = -.276, p = .001), IFN-γ (r = -.215, p = .007), IL17 (r = -.281, p = .001) and GM-CSF (r = -.227, p = .009). There were no correlations between change in cortisol and changes in IL-6 or MCP-1.

In addition to a decrease in cortisol (p < .001), there was also a decrease in both beta-endorphin (p < .001) and oxytocin (p < .001). Both neuropeptides had medium to large correlations with all cytokines (-3.67 < r < -.653, all p < .001) except MCP-1 for which there was a small correlation with beta-endorphin (r = -.286, p = .004) but no correlation with oxytocin. Both oxytocin and beta-endorphin were also positively correlated with cortisol (r = .427, p < .001 and r = .384, p < .001 respectively). Indeed, change in cortisol accounted for 20% of the variance in oxytocin levels (B = 0.965, SEM = .208, t_(93)_ = 4.641, p < .0001) and 17% of the variance in beta-endorphin levels (B = 0.589, SEM = 0.129, t_(120)_ = 4.562, p < .001) when adjusted for age and sex. Oxytocin and beta-endorphin were also both highly correlated with one another (r = .804, P < .001).

#### Between groups

Changes in a number of markers were consistent across the three groups. However, there were significant differences in response in three biomarkers: IL17, MCP1 and sTNFr1. Pairwise comparisons showed that there were significant increases in sTNFr1 in carers (p = .003) and bereaved carers (p < .001), but not in patients (p = .182). Pairwise comparisons also showed that bereaved carers only showed increases in MCP1 (p = .022) but carers did not show a significant increase (p = .567) and patients actually showed a decrease, although this did not reach significance (p = .166). Bereaved carers also showed the greatest IL17 responses with an increase across singing (p < .001). An increase was also seen for patients (p = .01) but not significantly for non-bereaved carers (p = .06).

### Psychobiological interactions

#### Across time

Change in the aggregate mood score was negatively correlated with post-intervention measures of IL-6, IL-17 and MCP-1 but none of the other cytokines. As all three biomarkers are often classed as pro-inflammatory markers, this demonstrates that improvements in mood were associated with lower levels of pro-inflammatory response but had no effect on anti-inflammatory response. In particular, a regression model adjusted for age and sex showed that mood explained 10% of the variance in MCP-1 (B = -2.215, SEM = 1.002, t_(134)_ = -2.211, p = .029) and once age and sex had been adjusted for, had a marginal effect on both IL-6 (R^2^ = .103, B = -1.797, SEM = 1.035, t_(181)_ = -1.737, p = .084) and IL-17 (R^2^ = .114, B = -0.958, SEM = 0.554, t_(173)_ = -1.728, p = .086). There were no associations between the aggregate stress score and pro-inflammatory levels or other biomarkers.

In addition, self-reported connectedness had a small positive correlation with beta-endorphin, accounting for 6% of the variation (B = 2.991, SEM = 1.107, t_(134)_ = 2.703, p = .008) and a near-significant correlation with oxytocin, accounting for 4% of the variation (B = 1.422, SEM = 0.773, t_(103)_ = 1.840, p = .069). No correlations were found between beta-endorphin, oxytocin and aggregate mood or stress scores.

## Discussion

The primary aim of this study was to explore whether group singing was associated with modulations in mood and neuroendocrine, neuropeptide and immune responses in three different groups affected by cancer who were regularly involved in choirs: carers, bereaved carers and patients. Our results demonstrated that a single hour of singing was associated with increases in positive affect, decreases in negative affect, a decrease in cortisol, beta-endorphin and oxytocin and a general activation of the cytokine network. The secondary aim was to explore differences in response between these four groups. Despite no baseline differences in response between the four groups, there were significant differences in response in three biomarkers: MCP1, IL17 and sTNFr1.

The main finding from this study is that singing was associated with a decrease in cortisol and increase in cytokine activity, found generally consistently across all five choirs and among all four groups. Previous studies involving singing have demonstrated that singing can modulate cortisol levels [8, 10]. However, this is the first study to demonstrate that singing is associated with modulation of cytokines, receptors and neuropeptides involved in immune response. These findings of a parallel decrease in cortisol and increase in cytokine activity in response to a short music intervention replicate those of a previous study involving making music in which mental health service users and carers involved in group drumming sessions had increases in IL2, IL4, IFNɣ and TNFα alongside decreases in cortisol, as in this study [27]. Furthermore, the same cortisol-cytokine negative relationship has been found in response to other psychosocial interventions in oncology [4–5]. One possible explanation for this response is that the reduction in cortisol following singing reduced glucocorticoid suppression of the immune system, leading to general activation of the cytokine network and increased immune activity [28]. Although, despite the correlations between cortisol and cytokines shown here, it is not possible to establish the direction of causality between the two.

Following on from this, there was also a pattern of a slightly weaker pro-inflammatory response compared with anti-inflammatory response among both carers and patients. There were no significant changes across time in the pro-inflammatory marker MCP-1, and similarly levels of IL-6 and IFN-γ reached significance using a p value of p < .05 but did not reach the adjusted significance value of p < .002. Furthermore, there was evidence that greater improvements in mood as a result of singing were associated with lower pro-inflammatory response. Interestingly, this appeared to be independent of stress levels. High levels of inflammation are associated with many mental health conditions including depression [29–30]. It is instructive to note that, among both patients and carers, those with the lowest levels of mental wellbeing and highest levels of depression experienced the greatest short-term improvement in mood across the singing session, and that these larger mood changes were associated with lower levels of inflammation. A hypothesis to be tested further is whether singing on a regular basis leads to larger and more sustainable improvements in mood and whether this affects inflammatory response.

Another finding was that singing was associated decreases in oxytocin and beta-endorphin. This is contrary to some previous research: two previous studies found that oxytocin increased in response to music [10, 31], although Kreutz’s study notably did not find decreases for cortisol, while Nilsson’s was in relation to listening to music and not singing. Similarly, the only previous study with beta-endorphin in response to listening to music found a reduction [32]. Oxytocin has been linked with social bonding and attachment, with higher oxytocin levels found, for example, in mothers and fathers in association with their social engagement with their children [33], and beta-endorphin is associated with feelings of euphoria [34]. However, both have also been found to be involved in stress regulation, with increased levels in response to a wide variety of stressful stimuli, serving to dampen blood pressure, heart rate and norepinephrine levels [35], [35–36]. Indeed, there have even been suggestions of beta-endorphin being regulated alongside the stress hormone adrenocorticotropin in stress response by the pituitary gland [37]. Given the correlations found between cortisol and both oxytocin and beta-endorphin and their correlations with one another, and the negative correlations between the neuropeptides and cytokines which mirrored the relationship between cortisol and the cytokines, it seems likely that the decrease found here was as part of a generalised down-regulation of stress response which may have over-ridden any social bonding or happiness-associated increase. Nevertheless, the positive associations found between changes in connectedness and beta-endorphin and near-significant associations with oxytocin may point to two contradictory processes at play.

In addition, this study examined differences in psychological and biological response between the three groups and as such whether singing is particularly effective for one group or equally beneficial for all. There were no significant differences in any of the psychological measures, suggesting that singing is similarly received by carers and patients. However, despite broadly similar responses in biomarkers, there were some discrepancies. Patients exhibited no significant changes in sTNFr1 response, whereas both carers and bereaved carers showed significant increases. TNF-α and its soluble receptor sTNFr1 have been implicated in cancer physiology as both promotors and inhibits of tumour progression. Some studies have found associations between heightened levels and increased risk of some cancers [38] as well as evidence that TNF-α is involved in tumorigenesis including cellular transformation, angiogenesis and metastasis [39]. However,

TNF-α can also induce cancer cell death [40]. Patients also showed opposite MCP1 responses to carers. MCP1 is a chemokine that recruits leukocytes including monocytes, dendritic cells and memory T cells to sites of inflammation [41], but can also be produced by cancer cells themselves and alter tumour behaviour [42]. The discrepancies in sTNFr1 and MCP1 activity in patients in response to singing, could be indicative that, although there may be broadly similar psychobiological pathways in response to singing, these responses are modulated by the specific immune profile of participants. However, the extent of this and the implications remain to be explored further. In addition, only bereaved carers showed significant responses in IL17 or MCP1, with no significant changes found for either carers or patients. Bereaved carers exhibited no baseline differences in mental health or either baseline levels or change in scores in mood or stress compared to current carers or patients, suggesting that this was not a difference in psychological processing. Instead it may be that current carers and patients had blunted IL17 and MCP1 responses. However, given this was a preliminary study, it would be premature to draw any firm conclusions from these findings, and they deserve to be studied more.

There were several limitations to the study. The main limitation is that the study was a preliminary uncontrolled study, and it is therefore possible that some of these psychological and biological results found would have occurred in the absence of singing, if participants had simply rested for 70 minutes. However, there is evidence that cytokines do not routinely change across an hour in the absence of an intervention [43–45]. Furthermore, participants’ stress levels were relatively low at the start of the session (23–33 on the visual analogue scale) suggesting that the findings were not wholly due to participants feeling acute stress at the start of the session that merely abated over the following hour. Nevertheless, a future controlled study is encouraged on the basis of these preliminary findings to explore these issues in more depth, and also explore which features of singing are most important (whether the social engagement of being in a choir, or the physical exercise of standing and singing for 70 minutes or the music itself). Second, we examined only the effects of a single session of singing. It remains unknown whether repeated singing sessions could impact on immune function and specifically if the results noted here could be maintained over time. Third, the study focused on participants who were current members of choirs. The ethos of the choirs was that no prior experience was necessary, so many participants had no singing experience prior to enrolment in the choirs. But it was nevertheless a self-selecting sample. It remains unknown whether singing could be of benefit for people who would not normally opt to be involved in a choir. Finally, cancer patients involved in the study had varying types of cancer and were at varying stages of treatment. This was a short intervention and confounding factors such as immune-affecting medication were removed to allow data to be analysed. However, it would be revealing to explore in more detail the effects of singing with defined patient groups and at targeted time points in cancer progression in order to confirm whether singing can have biologically meaningful effects.

## Conclusions

In conclusion, this study demonstrates associations between singing and reduced negative and increased positive effect, reduced cortisol, oxytocin and beta-endorphin and increased levels of cytokines. This is the first study to demonstrate the widespread immune effects of singing, in particular its effects on cytokines. Notably, the choice of biomarkers within this study included several that play important roles in cancer, including TNFα and the stem cell differentiator GM-CSF. Within the context of this preliminary study involving a single session of group singing, it is not possible to ascertain the implications of these changes, as they appear to occur as part of an acute non-specific cytokine activation. However, it would be of interest to ascertain whether such changes could be sustained with repeated exposure to the intervention over a longer time-span and with more specific patient groups. Such research could identify whether the psychosocial benefits of a communal activity such as group singing could lead to enhanced immune function in patients and carers affected by cancer.

## Conflicts of interest

Ian Lewis and Rosie Dow are employed by Tenovus Cancer Care who run the cancer choirs and funded the study. All other authors declare no conflicts of interest.

## Figures and Tables

**Table 1.** Demographics of participants.

	CARERS (n = 72)	BEREAVED CARERS (n = 66)	PATIENTS (n = 55)	F_df, df_	p
**Age (SD)**	56.86 (13.64)	59.69 (11.46)	60.81 (8.99)	1.91_2,186_	.151
**Sex (% women)**	80.6%	81.8%	80.0%	–	–
**Rehearsals attended (SD)**	44 (23)	46 (26)	44 (23)	0.11_2,190_	.892
**Psychological profile (SD)**					
HADSA	6.82 (4.08)	7.65 (3.71)	7.06 (4.27)	0.77_2,192_	.464
HADSD	3.25 (2.87)	3.74 (3.13)	4.22 (3.96)	1.35_2,192_	.261
CDRISC	47.89 (15.39)	49.09 (12.34)	48.27 (14.74)	0.38_2,191_	.686
WEMWBS	64.47 (24.55)	67.46 (19.65)	66.82 (17.87)	0.13_2,192_	.881
**Medication type (%)**					
None	37.5%	33.9%	60%	–	–
Hormonal	6.9%	12.8%	29.1%	–	–
Analgesic	5.6%	7.6%	5.5%	–	–
Antidepressant	4.2%	12.2%	3.6%	–	–
Antibody	–	–	1.8%	–	–
Cholesterol	4.2%	1.5%	0	–	–
Statins	4.2%	12.2%	1.8%	–	–
Blood pressure	15.3%	25.8%	3.6%	–	–
**Cancer type (%)**					
Breast	–	–	43.9%	–	–
Skin	–	–	17.1%	–	–
Gynaecological	–	–	14.6%	–	–
Prostate	–	–	7.3%	–	–
Thyroid	–	–	4.9%	–	–
Lymphoma	–	–	4.9%	–	–
Leukaemia	–	–	2.4%	–	–
Head/neck	–	–	2.4%	–	–
Bone	–	–	2.4%	–	–

**Table 2.** Psychological responses to singing in carers, bereaved carers and patients.

	CARERS		BEREAVED CARERS		PATIENTS		Time			Time*group		
	Mean (SEM)		Mean (SEM)		Mean (SEM)							
	Pre	Post	Pre	Post	Pre	Post	F_df, df_	p	Effect size (f)	F_3, 177_	p	Effect size (f)
**Mood scales**												
Afraid	13.5 (1.8)	6.5 (1.1)	11.3 (1.9)	6.3 (1.2)	10.2 (2.1)	7.8 (1.3)	34.96_1,187_	**<.001**	0.41	2.41_2,187_	.093	0.16
Angry	12.5 (1.8)	6.4 (1.5)	13.1 (1.9)	8.5 (1.6)	12.5 (2.1)	9.7 (1.7)	19.15_1,186_	**<.001**	0.32	0.86_2,186_	.423	0.10
Confused	12.0 (1.9)	7.5 (1.3)	9.6 (2.0)	7.0 (1.3)	16.7 (2.2)	8.9 (1.5)	23.82_1,185_	**<.001**	0.36	2.05_2,185_	.132	0.15
Energetic	49.6 (3.2)	77.0 (2.7)	49.5 (3.2)	70.6 (2.8)	47.5 (3.7)	72.6 (3.2)	140.44_1,186_	**<.001**	0.87	0.88_2,186_	.416	0.10
Happy	70.9 (2.7)	84.5 (1.9)	65.0 (2.8)	81.0 (1.9)	63.4 (3.1)	83.6 (2.2)	112.01_1,187_	**<.001**	0.77	1.44_2,187_	.239	0.12
Sad	16.1 (2.3)	7.1 (1.5)	20.5 (2.4)	10.5 (1.5)	14.9 (2.6)	9.4 (1.7)	40.78_1,184_	**<.001**	0.47	1.02_2,184_	.362	0.11
Tense	22.6 (2.7)	11.8 (1.9)	25.7 (2.8)	13.7 (1.9)	27.3 (3.2)	14.0 (2.2)	55.58_1,187_	**<.001**	0.54	0.19_2,187_	.831	0.04
Tired	37.7 (3.0)	19.9 (2.8)	43.6 (3.0)	25.7 (2.8)	41.4 (3.4)	28.8 (3.2)	64.92_1,185_	**<.001**	0.59	0.71_2,185_	.492	0.09
**Stress scales**												
Anxious	21.3 (3.0)	9.3 (1.8)	26.5 (3.1)	12.1 (1.8)	30.1 (3.5)	13.1 (2.1)	68.40_1,187_	**<.001**	0.61	0.65_2,187_	.522	0.08
Relaxed	66.9 (3.0)	85.3 (1.9)	63.5 (3.1)	81.4 (2.0)	65.8 (3.4)	82.0 (2.2)	110.31_1,187_	**<.001**	0.77	0.15_2,187_	.860	0.04
Stressed	23.8 (3.1)	11.1 (1.9)	32.5 (3.2)	14.3 (1.9)	29.9 (3.6)	11.8 (2.1)	88.02_1,187_	**<.001**	0.69	1.15_2,187_	.320	0.11
**Connectedness**												
Connected	71.9 (3.0)	89.9 (2.1)	66.4 (3.2)	80.4 (2.2)	72.2 (3.5)	85.5 (2.5)	107.72_1,186_	**<.001**	0.76	1.10_2,186_	.337	0.11
**Aggregate scales**												
Mood	75.6 (14.2)	87.8 (10.4)	73.8 (14.7)	85.0 (10.2)	73.5 (13.2)	84.8 (11.4)	162.10_1,187_	**<.001**	0.93	0.13_2,187_	.880	0.03
Stress	26.1 (19.2)	11.7 (11.7)	31.8 (22.1)	15.0 (14.3)	31.4 (21.6)	14.3 (12.9)	144.83_1,187_	**<.001**	0.88	0.45_2,187_	.640	0.07

**Table 3.** Biological responses to singing in carers, bereaved carers and patients.

	CARERS		BEREAVED CARERS		PATIENTS		Time			Time*group		
	Mean (SEM) pg/ml		Mean (SEM) pg/ml		Mean (SEM) pg/ml							
	Pre	Post	Pre	Post	Pre	Post	F_df, df_	p	Effect size (f)	F_3, 177_	p	Effect size (f)
**Cytokines**												
GM-CSF	2.55 (0.12)	2.88 (0.12)	2.83 (0.13)	3.12 (0.12)	2.77 (0.14)	3.00 (0.13)	32.14_1,161_	**<.001**	0.45	0.38_2,161_	.686	0.07
IFNg	4.23 (0.16)	4.27 (0.14)	4.05 (0.17)	4.45 (0.15)	4.39 (0.19)	4.53 (0.17)	4.82_1,184_	**.029**	0.16	1.59_2,184_	.208	0.13
IL-17	4.06 (0.19)	4.40 (0.19)	3.64 (0.21)	4.66 (0.21)	3.86 (0.22)	4.40 (0.22)	31.54_1,171_	**<.001**	0.43	3.33_2,171_	**.038**	0.19
IL-2	3.32 (0.11)	3.67 (0.10)	3.48 (0.12)	3.78 (0.11)	3.39 (0.13)	3.69 (0.12)	41.16_1,178_	**<.001**	0.48	0.10_2,178_	.903	0.03
IL-4	2.48 (0.08)	2.68 (0.08)	2.51 (0.09)	2.77 (0.09)	2.60 (0.09)	2.79 (0.09)	33.31_1,175_	**<.001**	0.44	0.39_2,175_	.677	0.06
IL-6	2.62 (0.11)	2.78 (0.97)	2.53 (0.12)	2.70 (0.10)	2.75 (0.13)	2.79 (0.11)	5.33_1,185_	**.022**	0.17	0.53_2,185_	.590	0.08
MCP-1	6.19 (0.12)	6.27 (0.14)	5.85 (0.12)	6.19 (0.14)	6.09 (0.14)	5.85 (0.16)	0.46_1,128_	.501	0.06	3.28_2,128_	**.041**	0.23
TNF-a	2.84 (0.13)	3.09 (0.13)	2.70 (0.14)	3.12 (0.14)	2.63 (0.16)	3.00 (0.15)	32.95_1,185_	**<.001**	0.42	0.81_2,185_	.445	0.10
**Receptors**												
sIL-2ra	4.27 (0.14)	4.47 (0.13)	4.24 (0.15)	4.68 (0.14)	4.41 (0.16)	4.71 (0.15)	21.10_1,152_	**<.001**	0.37	1.09_2,152_	.340	0.12
sTNFr1	4.62 (0.14)	4.95 (0.14)	4.69 (0.15)	5.26 (0.15)	4.80 (0.16)	4.97 (0.16)	28.71_1,182_	**<.001**	0.40	3.13_2,182_	**.046**	0.18
**Neuropeptides**												
β-endorphin	4.79 (0.23)	4.20 (0.24)	4.57 (0.24)	3.79 (0.24)	4.55 (0.27)	4.11 (0.28)	23.36_1,135_	**<.001**	0.42	0.58_2,135_	.561	0.10
Oxytocin	5.14 (0.32)	3.74 (0.31)	5.15 (0.35)	3.88 (0.35)	4.45 (0.36)	3.62 (0.35)	33.19_1,103_	**<.001**	0.57	0.72_2,103_	.490	0.12
**Glucocorticoids**^a^												
Cortisol	2.73 (0.14)	2.18 (0.13)	2.96 (0.16)	2.45 (0.15)	2.91 (0.17)	2.47 (0.15)	48.46_1,156_	**<.001**	0.56	0.19_2,156_	.831	0.04
